# Concomitant Deep Venous Thrombosis, Femoral Artery Thrombosis, and Pulmonary Embolism after Air Travel

**DOI:** 10.1155/2014/174147

**Published:** 2014-08-21

**Authors:** Salim Abunnaja, Marshall Clyde, Andrea Cuviello, Robert A. Brenes, Giuseppe Tripodi

**Affiliations:** The Stanley J. Dudrick Department of Surgery, Saint Mary's Hospital, 56 Franklin Street, Waterbury, CT 06706, USA

## Abstract

The association between air travel and deep venous thrombosis and/or pulmonary embolism “economy-class syndrome” is well described. However, this syndrome does not describe any association between long duration travel and arterial thrombosis or coexistence of venous and arterial thrombosis. We present a case of concomitant deep venous thrombosis, acute femoral artery thrombosis, and bilateral pulmonary embolisms in a patient following commercial air travel. Echocardiogram did not reveal an intracardiac shunt that may have contributed to the acute arterial occlusion from a paradoxical embolus. To our knowledge, this is the first report in the literature that associates air traveling with both arterial and venous thrombosis.

## 1. Introduction

The association between air travel and deep venous thrombosis/pulmonary embolism was first reported in 1954 [1]. Soon after, the phrase “economy-class syndrome” [2, 3] was used to describe this problem, with several more published case series appearing in the literature [4–6]. The mechanism behind the increased risk for clotting complications was proposed to include blood stasis, along with one or more of the following: lower extremity fluid retention, hemoconcentration of clotting factors, and possible activation of the coagulation cascade [7–9]. A few authors have also related this condition to long duration travel by car and train [10–12]. This syndrome, however, does not describe an association between long duration travel and arterial thrombosis or the coexistence of venous and arterial thrombosis simultaneously. We report a case of concomitant deep venous thrombosis and acute femoral artery thrombosis along with bilateral pulmonary embolism after a long overseas flight.

## 2. Case Report

A 50-year-old woman from Montenegro presented to our institution with shortness of breath that was associated with pleuritic chest pain. Her symptoms began during a long overseas flight and progressively worsened after disembarking the plane.

**Figure 1:**
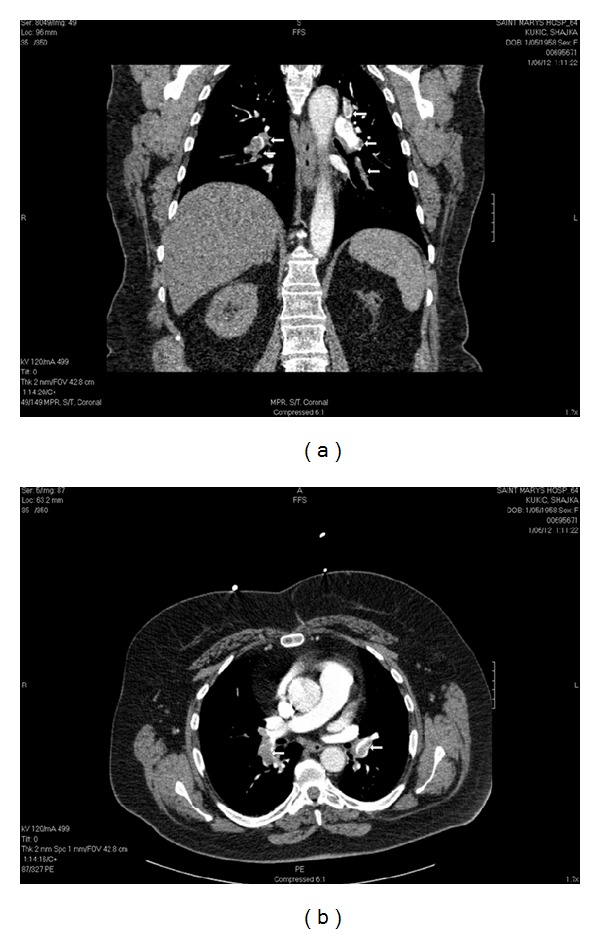
CT scan of chest, coronal view. White arrows point to bilateral pulmonary emboli.

She also complained of left leg pain and swelling. The patient's past medical history was significant for hypertension and diabetes mellitus. She never smoked and was not on any hormonal treatments. On physical exam, heart rate was 115, respiratory rate was 32, and she appeared to be in mild distress. Bilateral breath sounds were clear to auscultation. Of note, the patient had swelling of the left lower extremity and foot with some dark discoloration, absence of distal pulses, and impaired sensation. Initial laboratory tests revealed respiratory alkalosis (pH 7.45, pCO2 28, paO2 111, and HCO3 22.5), a normal coagulation profile, and a D-dimer of 1300 ug/L. Cardiac enzymes were within normal limits. EKG showed sinus tachycardia. A CT scan of the chest revealed bilateral pulmonary embolisms (Figure 1). Arterial and venous Doppler imaging of both lower extremities showed an occlusive left popliteal vein thrombus and an occlusive left common femoral artery thrombosis.

**Figure 2:**
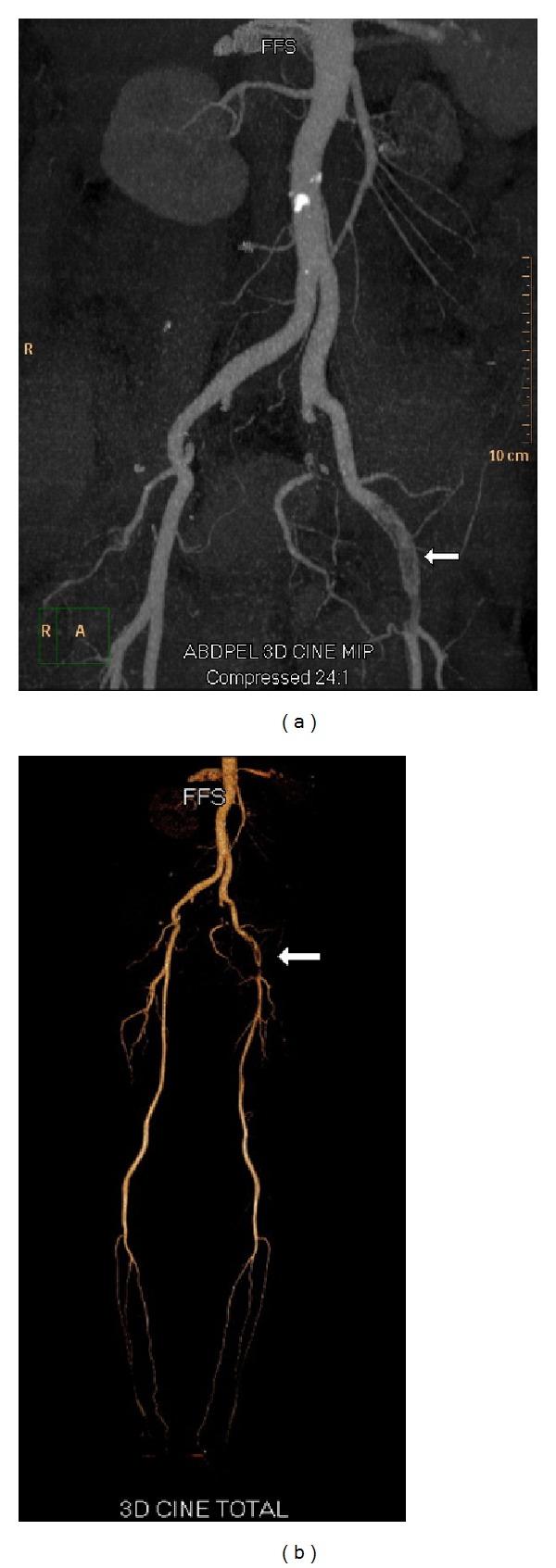
CTA of bilateral lower extremities. White arrow points to filling defect of left external iliac artery.

**Figure 3:**
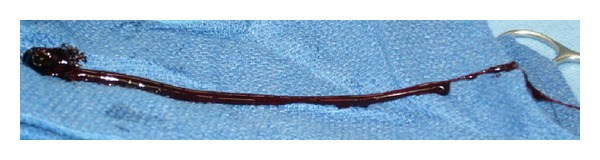
Large blood clot removed from the left iliofemoral artery intraoperatively. CTA of bilateral lower extremities. White arrow points to filling defect of left external iliac artery.

A heparin drip was initiated immediately and the patient was admitted to the intensive care unit with a low threshold to proceed to the operating room for either thrombectomy or catheter-based thrombolysis. Fortunately, few hours following the initiation of treatment a significant improvement was noticed in the patient's shortness of breath and chest pain. Additionally, her left leg swelling and skin discoloration were markedly improved as well; however, she continued to have a dull aching pain in her left foot with weak, monophasic, dopplerable distal pulses. The patient underwent a CTA of her left lower extremity with distal runoff, which demonstrated a subocclusive filling defect extending from the origin of the left common femoral artery to the distal superficial femoral artery, with normal popliteal and three-vessel runoff to the ankle (Figure 2). With an unclear source of this arterial thrombosis, an echocardiogram was obtained to rule out a cardiac source of an acute embolus, as well as a paradoxical systemic arterial embolism through a patent foramen ovale (PFO). The transthoracic echocardiogram was normal with no evidence of an intracardiac shunt, right heart strain, or mural thrombus. Given the acute nature of the patient's condition and her only partial response to nonoperative management, the patient was taken to the operating room. An open mechanical left iliofemoral arterial thrombectomy was performed using a Fogarty catheter. A large clot was retrieved from both the superficial femoral and external iliac arteries (Figure 3). Postoperatively the patient's symptoms dramatically improved and her physical exam revealed palpable distal pulses in her left foot with mild reperfusion symptoms.

Although the initial results for hypercoagulation workup such as protein C, protein S factor II assay, and ANA screen were all within normal limits, these results are difficult to interpret in the setting of acute thrombosis and anticoagulant medication therapy. She was discharged on Warfarin with a plan for full hypercoagulation workup after discontinuing the Warfarin in 6 months with a potential need for long life anticoagulation pending the workup. After discharge, the patient traveled back to her homeland and was unfortunately lost for followup.

## 3. Discussion

Stasis caused by sitting and immobility during prolonged journeys (>5 hours) is considered a risk factor for deep venous thrombosis and pulmonary embolism. Rudolf Virchow described a triad that predisposes an individual to thrombosis, which includes immobility, endothelial damage, and hypercoagulability. This case report hones in on the immobility aspect experienced by long distance travelers but can also include patients who are immobile due to disabilities or recent major surgery. Endothelial damage can be caused by a number of variables including smoking, atherosclerosis, trauma, and even prolonged immobility [7, 13, 14]. A hypercoagulable state can be induced, as with patients with cancer, or inherited, like those individuals with factor 5 Leiden or protein C/S deficiencies [14]. What is remarkable is that over the last twenty years, greater than 200 cases of pulmonary embolism have been reported in association with “economy-class syndrome” [2, 7, 11, 13, 15]. Landgraf et al. proposed the mechanism mentioned previously of blood stasis in association with immobility effects such as fluid retention in the legs [9], reduction of oxygen in the cabin [16], hemoconcentration secondary to dehydration [14], and activation of coagulation [17]. It should be noted that this syndrome has also been described in first class or business class passengers and even in prolonged overland journeys like those via train, car, or coach [10–12]. For this reason, the syndrome has been referred to by some as “travelers' syndrome” [18]. In March 2001, the World Health Organization (WHO) accepted that there was a probable risk of presenting with pulmonary embolism after prolonged flights despite the low incidence and the presence of other risk factors in most of the passengers affected.

Acute arterial thrombosis on the other hand is traditionally regarded as a different disease with respect to pathophysiology, epidemiology, and treatment strategies when compared to venous thrombosis. To our knowledge, this is the first report in the literature that associates air travel with both acute venous and arterial thrombosis. Arterial thrombi tend to occur at places where plaques are formed and where shear stress is high, which results in platelet rich “white thrombi” [19]. In contrast, with venous thrombotic disease, thrombi tend to occur at sites where the vein wall is undamaged and blood flow and shear stress are low, resulting in red cell-rich “red thrombi” [19]. Stasis caused by sitting and immobility during prolonged traveling is therefore not considered a risk factor for acute arterial thrombosis. Nevertheless, it appears that venous thrombosis and arterial thrombosis are not completely separate entities. Becattini et al. demonstrated a 40% decrease in DVT recurrence rate by initiating antiplatelet therapy after cessation of warfarin therapy for DVT [20]. Recent research has shown a 40 to 50% risk reduction for venous thrombosis occurrence in patients taking statins for arterial diseases [21]. On the other hand, there is also a 1.5- to 3-fold increased venous thrombotic risk in individuals who have been exposed to traditional arterial thrombotic risk factors like diabetes, hypertension, and dyslipidemia [21, 22]. Furthermore, it appears from the literature that patients with arterial thrombosis have from 1.2-fold to more than 4-fold increased risk of developing subsequent venous thrombosis [23]. Despite these associations, acute cases of simultaneous arterial and venous thromboses are rarely seen in clinical practice, and there have only been a few cases reported in the literature [24–27], none of which have been linked to air travel.

It is important not to forget about a possible PFO in patients presenting with the coexistence of pulmonary and paradoxical systemic arterial embolism [28]. A small PFO is usually hemodynamically insignificant, while large-diameter PFOs may act as a pathway for the passage of thrombi, air, fat, vegetations, or vasoactive substances from the venous to the arterial circulation, potentially causing paradoxical emboli and stroke [29]. In our case, a normal echocardiogram excluded PFO as a possible cause of this unusual coexistence of pulmonary and systemic thrombosis.

Regardless of patient history or risk factors, as some patients may carry a coagulation disorder that has thus far been silent, there are certain precautions that those embarking on a long, sedentary journey can take to avoid coagulation complications such as deep venous thrombosis and pulmonary embolism. These recommendations include avoiding sitting with crossed legs, attempting to stand or move about every two hours for a couple of minutes, and engaging in flexion-extension exercises while seated. The avoidance of dehydration, excessive alcoholic intake, and tight clothing can assist in decreasing the risk for vasoocclusive complications during long journeys.

In this case, long-duration air travel in a seated position likely caused venous stasis leading to deep vein thrombosis, and we speculate it may have caused prolonged subtotal arterial compression which may have predisposed the patient to arterial thrombus formation. Due to the patient being lost to followup, we cannot investigate a possible hypercoagulable state which to this point has been unidentified. Case reports have attributed acute limb arterial thrombosis to a known hypercoagulable state (inherited [30] or acquired [31]) although the literature has not shown there be a statistical association between the two. In this case and similar cases, a hypercoagulable workup should include testing for Factor V Leiden and prothrombin 20210 mutations, deficiency of protein C, protein S, and antithrombin III; elevation of clotting factors VIII, IX, XI, and fibrinogen and homocysteine levels; and testing anticardiolipin antibodies. Most clinicians will elect to do the workup two to four weeks after stopping anticoagulation, because the results of some of these tests may potentially be affected by acute thrombosis and anticoagulation. The possibility of an unidentified cancer as a cause of the hypercoagulable state should also be kept in mind and investigated.
